# Predicting Metabolism from Gene Expression in an Improved Whole-Genome Metabolic Network Model of *Danio rerio*

**DOI:** 10.1089/zeb.2018.1712

**Published:** 2019-08-01

**Authors:** Leonie van Steijn, Fons J. Verbeek, Herman P. Spaink, Roeland M.H. Merks

**Affiliations:** ^1^ Mathematical Institute, Leiden University, Leiden, Netherlands.; ^2^ Leiden Institute of Advanced Computer Science, Leiden University, Leiden, Netherlands.; ^3^ Institute of Biology Leiden, Leiden University, Leiden, Netherlands.

**Keywords:** metabolism, metabolic modeling, *Mycobacterium marinum*, tuberculosis, genome-scale metabolic model, flux-balance analysis

## Abstract

Zebrafish is a useful modeling organism for the study of vertebrate development, immune response, and metabolism. Metabolic studies can be aided by mathematical reconstructions of the metabolic network of zebrafish. These list the substrates and products of all biochemical reactions that occur in the zebrafish. Mathematical techniques such as flux-balance analysis then make it possible to predict the possible metabolic flux distributions that optimize, for example, the turnover of food into biomass. The only available genome-scale reconstruction of zebrafish metabolism is ZebraGEM. In this study, we present ZebraGEM 2.0, an updated and validated version of ZebraGEM. ZebraGEM 2.0 is extended with gene-protein-reaction associations (GPRs) that are required to integrate genetic data with the metabolic model. To demonstrate the use of these GPRs, we performed an *in silico* genetic screening for knockouts of metabolic genes and validated the results against published *in vivo* genetic knockout and knockdown screenings. Among the single knockout simulations, we identified 74 essential genes, whose knockout stopped growth completely. Among these, 11 genes are known have an abnormal knockout or knockdown phenotype *in vivo* (partial), and 41 have human homologs associated with metabolic diseases. We also added the oxidative phosphorylation pathway, which was unavailable in the published version of ZebraGEM. The updated model performs better than the original model on a predetermined list of metabolic functions. We also determined a minimal feed composition. The oxidative phosphorylation pathways were validated by comparing with published experiments in which key components of the oxidative phosphorylation pathway were pharmacologically inhibited. To test the utility of ZebraGEM2.0 for obtaining new results, we integrated gene expression data from control and *Mycobacterium marinum*-infected zebrafish larvae. The resulting model predicts impeded growth and altered histidine metabolism in the infected larvae.

## Introduction

The zebrafish (*Danio rerio*) has become a widely used model organism for the study of vertebrate metabolism.^1,2^ Its genome has been sequenced and annotated^3^ and the CRIPSR-Cas technique has made it easier than ever to study the role of specific metabolic genes.^4^ For example, zebrafish have been used to test the toxicity of drugs on liver metabolism and the effect of liver metabolism on internal drug concentration.^5^ Zebrafish have also been used in studies of metabolic diseases such as diabetes, obesity, and fatty liver disease, often combining sequencing with visualization of gene expression.^1^

Mathematical and computational techniques make it possible to use such metabolic gene expression data to predict the flux of metabolites through single cells or even whole organisms. Genome-scale metabolic reconstructions, or metabolic maps for short, are models that consist of two parts: a metabolic network of the organism and the genes underlying this network. This network reconstruction is based on the genes coding for metabolic proteins present in the genome and sometimes requires manual curation to fills in gaps in the network.^6^

Metabolic maps make it possible to predict how metabolites flow through a network of biochemical reactions, finally resulting in resources for growth or the availability of energy. Because in one network, an infinite number of alternative flow distributions are equally likely, a sensible prediction can only be made under the assumption of an *objective*, for example, optimal biomass production or optimal production of ATP, and a number of *constraints* on the possible fluxes. Most techniques assume flux balance, meaning that all biochemical concentrations are in equilibrium. Additional constraints can be given by known or assumed concentrations of enzymes, leading to a maximum flux through the reaction.

Mathematical techniques to make these predictions include Flux-Balance Analysis (FBA)^7^ and derivate methods as Flux Variance Analysis,^8^ Minimization of Metabolic Adjustment,^9^ and Expression flux.^10^ These predict the production rate of biomass or of a certain metabolite, for a given substrate, and sometimes supplemented with expression data. These predictions are valuable for finding suitable substrates for microorganism-based production in bioreactors. Another feature of these methods used to predict the flux through genome-scale metabolic models is the ability to study the effects of gene knockouts or gene expression on metabolism by constraining or removing reactions in the reaction network.^11,12^ This gives insight into the metabolic routing or rerouting of an organism and can be helpful in acquiring the aspired phenotype of an organism, but it can also give insight into the metabolic fluxes of different cell types.

With the increasing presence of metabolic data of healthy and diseased zebrafish, and the availability of genetic data, a genome-scale metabolic model of the zebrafish is tremendously useful. So far, genome-scale metabolic models have been proposed mainly for single-cell model organisms, such as *Escherichia coli* and *Saccharomyces cervesiae*,^13–15^ as well as pathogens such as *Salmonella typhimurium*^16^ and *Mycobacterium tuberculosis*.^17^ For these unicellular organisms, very accurate growth predictions have been made. Multicellular organisms, particularly vertebrates, are less well represented in the list of genome-scale metabolic models. So far, reconstructions have been made for human,^18^ mouse,^19^ Chinese hamster,^20^ fish,^21,22^ and recently, rat.^23^ Whole-organism modeling is less common for these multicellular organisms, as metabolic functions are distributed over different tissues. However, modeling specific cell types has been done, such as erythrocytes^24^ and cancer cell lines,^25^ as well as integrating different cell types into a larger model, such as a combined model, including adipocytes, myocytes, and hepatocytes.^26^

Why do we require a specific zebrafish genome-scale metabolic reconstruction when other vertebrate models exist? Despite the high metabolic similarity to human and mouse, there are subtle differences between zebrafish metabolism and the metabolism of these mammals that affect their required nutrients. For example, inositol-3-phosphate synthase is an enzyme present in humans and mice, but it is absent in zebrafish, preventing it from converting glucose-6-phosphate into inositol 3-phosphate.^27^ This makes inositol an essential nutrient for zebrafish.

The difference in metabolism aside, the main reason to make a specific zebrafish genome-scale metabolic model is the genomic structure. The teleost lineage underwent a whole-genome duplication event after the radiation from their common ancestor with mammals, which resulted in numerous genes still having duplicate copies compared to mammals.^28^ As a result, there are more paralogous genes in the zebrafish genome than in mammals. Hence, if one wants to study the effects of genes on metabolism, translating a human or mouse genome-scale metabolic reconstruction into a zebrafish specific model by orthologous genes is not sufficient. Foremost, this translation is hampered by these paralogs as it does not make the translation one-to-one, and furthermore, many paralogs have evolved different subfunctions, increasing the functional difference between the zebrafish paralogs and the human or mouse orthologs. So to model the effects of genes on zebrafish metabolism, a zebrafish-specific genome-scale model is necessary.

Existing genome-scale models for zebrafish are MetaFishNet^21^ and ZebraGEM.^22^ MetaFishNet is a metabolic model derived from the genome of multiple fish species, including zebrafish, and focuses on individual pathways. As these pathways are not interconnected or divided into cell compartments, MetaFishNet is not suitable for whole-cell or whole-organism modeling using Flux Balance Analysis (FBA) methods, and therefore functions mainly as a reference tool, instead of a simulation tool. The fact that it combines multiple fish genomes also makes it harder to compare insights gained from this model to *in vivo* experimental results, as some pathways are solely based on the genome of one of those five fish species and do not occur in the other four fish species.

The other model, ZebraGEM, is based on the zebrafish genome and is a whole-cell and compartmentalized reconstruction. It contains 2911 reactions, of which 2446 are gene-associated reactions based upon 1498 genes and can be used for whole-cell metabolism modeling. It was reported to fulfill a list of 160 metabolic functions, such as the production of amino acids and biosynthesis and degradation of secondary metabolites. The model also predicted that the synthesis of taurine is through a metabolic pathway dependent on cysteine sulfinic acid decarboxylase, which is in line with experimental findings.^29^

Currently, ZebraGEM cannot be used for modeling large screens of single gene knockouts or for the integration of gene expression data, as it lacks GPR. GPRs describe how gene products associated to a reaction work together, that is, whether they form a complex enzyme, are isoenzymes, or a combination of these. They provide a logical framework to decide whether a reaction can take place when one or more of its underlying genes are knocked out, and hence, they are of great importance when it comes to modeling gene knockouts.

In this article, we describe the modifications applied to ZebraGEM to fit our modeling needs and to fit standards of genome-scale metabolic reconstructions, as well as demonstrate a number of ways in which the updated model can be used. Briefly, the modifications fall into three categories. First, we added the GPRs, to facilitate gene knockout and gene expression modeling. Second, we renamed components of the model according to BiGG Models standard names,^30^ to ease comparison with genome-scale metabolic reconstructions of other organisms. Finally, we extended the model with essential reactions for pathways already present, or changed the reversibility of reactions already present in the model.

We have validated the renewed model against the metabolic functions the original model was reported to fulfill. Using the updated model, we predicted a minimal feed composition and were able to make predictions of mitochondrial function with respiration simulations. Finally, we also proved the usefulness of the newly added GPRs: we performed a large single-knockout and double-knockout screening and predicted lethal knockouts, and we also integrated gene expression data with the model to predict metabolic differences between control zebrafish larvae and larvae infected with *Mycobacterium marinum*.

## Methods

**FIG. 1.**
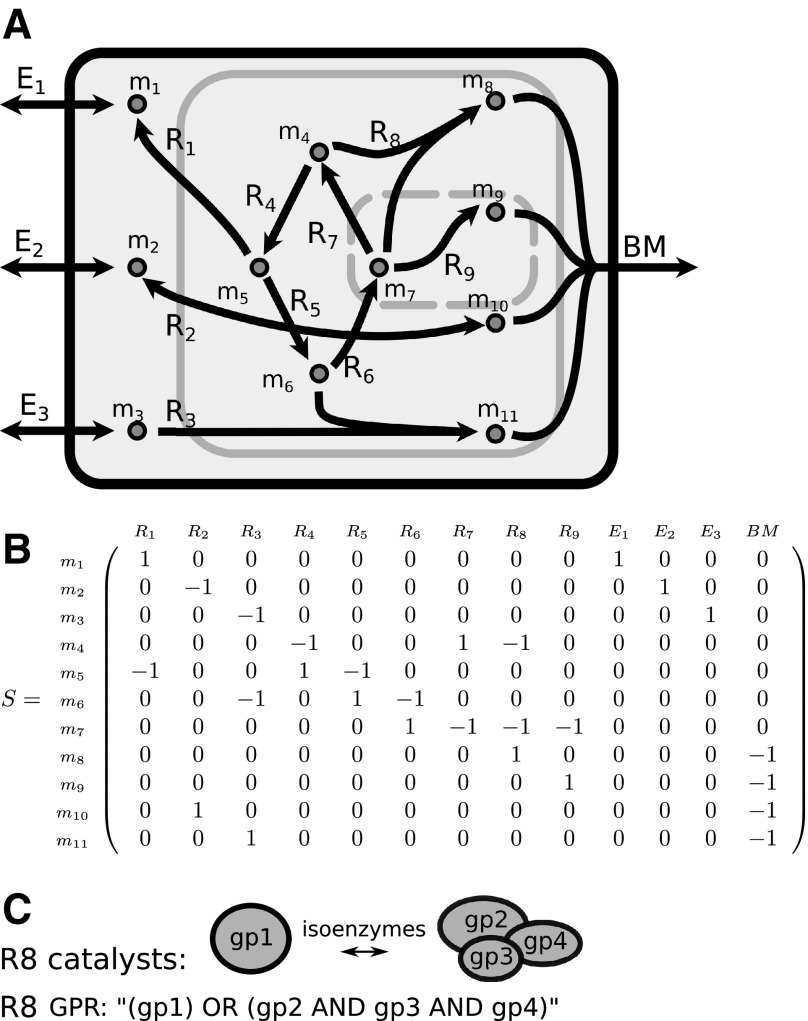
Important components of a genome-scale metabolic reconstruction are the metabolic network **(A, B)** and the GPR **(C)**. **(A)** Graphical overview of a simplified metabolic network. Reactions within the *black border* are part of the system and hence have mass balance. The *solid gray border* indicated the cell membrane and the *dashed gray border* indicates cell organelle membranes. Reactions E1–E3 are exchange reactions and are not mass balanced, allowing for import and export of metabolites. Reaction BM is a biomass reaction, taking biomass precursor metabolites and exporting them to biomass; **(B)** stoichiometric matrix representation of the network shown in **(A)**; **(C)** example of how isoenzymes and protein complexes are translated into a GPR. GPR, gene-protein-reaction associations; gpx, gene product x.

The genome-scale metabolic reconstruction (“metabolic map”) of zebrafish consists of the following: (1) a metabolic network describing the reactions that can occur in the organism and (2) the genes that are associated with those reactions (Fig. 1). The network on its own can be used for modeling metabolism, and the associated genes give extra handles to this modeling. In this section, we give a general overview of the metabolic network component and gene component of a genome-scale metabolic reconstruction, as well as describe the modeling method called FBA. We also briefly address the representation of this model in a computer file.

### Metabolic network

The metabolic network part of a metabolic map can be represented by a matrix *S* (Fig. 1A, B). This matrix contains the ratio between reactants and products, or stoichiometry, for each reaction within the network, and is called a stoichiometric matrix. The rows represent the metabolites and the columns represent the reactions. The coefficient at the intersection of a specific row and column indicates the contribution of that metabolite to that reaction. Some of the reactions are of a special type, the so-called exchange reactions. These exchange reactions either have only a reactant or only a product, and hence do not preserve mass. They represent the influx and efflux of metabolites in and out of the system.

### Flux Balance Analysis

The standard method for constraint-based metabolic modeling is FBA.^7^ For a given metabolic network and a given objective function, FBA computes the optimal flux through the metabolic network that minimizes or maximizes the objective function. The first assumption upon which FBA is based, is that an organism will adjust its fluxes such that the internal metabolites, indicated with *c*, are in equilibrium, that is

\begin{align*}
\begin{matrix} \displaystyle { { \frac { dc }  { dt } } = S \cdot \vec f = 0 } \\ \end{matrix} \tag { 1 }
\end{align*}

with $\vec f$ the vector representing the fluxes of the reactions in the metabolic network. Some of these fluxes can be constrained. For example, exchange reactions can be constrained due to limited availability of the exchanged metabolite in the environment. Also, irreversible reactions can be constrained, as they cannot have a negative flux. This can be formulated as follows:

\begin{align*}
\begin{matrix} {{a_i} \le {f_i} \le {b_i}} \\ \end{matrix} \tag{2}
\end{align*}

with *a_i_* and *b_i_* indicating the lower bound and upper bound of the flux of reaction *i*. Sometimes an exchange reaction has a strictly positive lower bound, indicating that the system should at least produce that amount of the exchanged metabolite. These reactions are called demand reactions.

Solving Equations 1 and 2 together can lead to an infinite number of solutions. Within this solution space, FBA selects for a smaller solution space based on a predefined *objective*, for example, that the organism optimizes its metabolic fluxes for a specific reaction or for biomass production. This optimized reaction, or objective function *f_obj_*, can be any reaction in the metabolic network, but most often, it is a biomass function. The biomass function lists all the precursor metabolites and energy-carrying metabolites required for the accumulation of biomass. Unless stated otherwise, we will use the biomass function as the objective function. The full formulation of the FBA problem then becomes as follows:

Optimize

\begin{align*}
\begin{matrix} {{f_{obj}} \;} \\ \end{matrix} \tag{3}
\end{align*}

such that,

\begin{align*}
S \cdot \vec f = 0 , {a_i} \le {f_i} \le {b_i}
\end{align*}

This forms a linear programming problem and can easily be solved using linear programming solver software, for example, GNU linear programming kit (GLPK) or Gurobi. In this work, we have used CPLEX IBM ILOG CPLEX.

Once the linear programming problem is solved, the solution $\vec f$ gives a flux distribution of the metabolic network for the given constraints. This gives insight into which pathways are used and their relative contribution can be computed. By changing the upper and lower bounds in Equation 2, one can test the flux distribution in different scenarios, such as comparing the growth rate under different sets of substrates.

Some common variations on FBA are parsimonious FBA^31^ (pFBA) and Flux Variability Analysis (FVA),^8^ which are multiobjective linear programming problems. After solving the original FBA problem, they then optimize a second objective. For pFBA, the secondary objective is to minimize the total sum of fluxes, that is, $\min \sum \left\vert {{f_i}} \right\vert$, while maintaining the same constraints as in the FBA problem, together with keeping the previous objective ${f_{obj}}$ at its optimum. FVA is a method that explores more of the solution space, by searching for the minimum and maximum flux of each reaction. So after doing FBA, a new linear programming problem first minimizes and then maximizes each *f_i_*, while also maintaining ${f_{obj}}$ at its optimum and regarding all the previous constraints.

Multiple software packages for FBA exist. These function as an interface between the user and the linear programming solver. They allow for easy manipulation of bounds, easy addition and removal of reactions in the metabolic network, and modification of the GPRs, without having to keep track of the linear programming problem manually. The software used in this study is CobraPy,^32^ combined with the CPLEX solver.

### Genes and constraint-based modeling

The second part of the metabolic map consist of the associated genes. These genes, responsible for the enzymatic reactions in the metabolic network, are represented using GPR. In its simplest form, the GPR links each enzyme with a biochemical reaction. If two enzymes catalyze the same reaction, the GPR becomes a logical expression. If they are isoenzymes, for example, they can both independently catalyze the reaction, an “OR” function is used. If the two enzymes form a complex such that both must be present to catalyze the reaction, an “AND” function is used. More complex GPRs can be described by nested logical expressions (Fig. 1C). In case multiple, equivalent logical expressions are possible, the disjunctive normal form is used, that is, a summation of all possible isoenzymes.

Using the GPRs, gene knockouts or gene expression data can be integrated into constraint-based models. A standard way of integrating gene knockouts is to set each occurrence of the knocked-out gene in a GPR to False and evaluate the GPRs. If any of these GPRs also evaluates to false, then constrain the corresponding reaction to 0 flux by setting its upper and lower bound to 0. Gene expression data can be integrated into constraint-based modeling in alternative ways.^33–36^

Although details vary, these methods either penalize fluxes over reactions with no or low expression and minimize the penalty or they set the lower and upper bound of fluxes depending on the expression level. The gene expression data integration method used in this study is Gene-centric flux (GC-flux).^37^ In this study, the linear programming problem is slightly altered from the original stoichiometric matrix-based linear programming problem. Using the GPRTransform package,^38^ we split up each reaction into multiple versions of the same reaction, one for every possible isoenzyme. The sum of the fluxes of all the reactions containing a certain gene in their GPR is then constrained by the expression level of that gene. Although many choices exists for how the expression level gives an upper bound, the simplest one is to take the expression level itself. So if we rephrase Equation 3 with the altered stoichiometric matrix $S \prime$, the new programming problem becomes as follows:

First optimize

\begin{align*}
\begin{matrix} {{f_{obj}} \;} \\ \end{matrix} \tag{4}
\end{align*}

such that,

\begin{align*}
S \prime \cdot \vec f = 0 , {a_i} \le { \vec f_i} \le {b_i}
\end{align*}

\begin{align*}
\mathop \sum \limits_{r \in {R_g}} \left\vert {{{ \vec f}_r}} \right\vert \; \;  \le  \;{E_g} \;  \; \; \forall g \in {G}
\end{align*}

Here *R_g_* denotes the reactions belonging to gene *g*, *E_g_* the expression of that gene, and *G* the total gene set. Basically, this algorithm distributes the gene expression among the different enzyme complexes, and hence the related reactions, of that gene, assuming that each molecule of a gene product can only take part in one complex at a time.

The GC-flux algorithm originally also minimized the length of the flux vector, to obtain the most parsimonious flux distribution that optimizes the objective. We did not minimize the flux vector length, but applied FVA together with computing the relative flux range change (RFRC) to compare between the different gene expression data sets. With FVA, we determine for each *f_i_* its minimum and maximum value that still allow for the objective to be optimized. To compare the flux ranges between different conditions, we compute the RFRC of reaction *i* as follows^39^:

\begin{align*}
RFR { C_i } = { \frac { { c_ { 2 , i } } - { c_ { 1 , i } } }  { \frac { 1 }  { 2 } \left( { { r_ { 2 , i } } + { r_ { 1 , i } } } \right) } } \; ,
\end{align*}

with ${c_{n , i}}$ the center ($\frac { 1 }  { 2 } ( { f_ { i , max } } + { f_ { i , min } } )$) of the flux range of reaction *i* in condition *n*, and ${r_{n , i}}$ the range width $( {f_{i , max}} - {f_{i , min}} )$.

### Data standards for representation of metabolic maps

To facilitate exchange of computational models, such as metabolic models, in systems biology, the Systems Biology Markup Language (SBML) has been developed.^40^ Different elements of a metabolic map, such as metabolites, reactions, genes, and GPRs, are represented by their own class in SBML. For this, we use the fbc package, the Flux Balance Constraints extension of SBML. This package is especially designed to describe these genome-scale metabolic reconstruction elements, and has specified guidelines on how an entity should be represented in an SBML file.^41^ The original model was already an SBML file, but predates the fbc package's release. Therefore, we adapted the model to fit with the fbc package guidelines.

#### Metabolite, reaction, and gene nomenclature

Aside from the file structure, there are also standards for the names of metabolites and reactions. This facilitates comparison and interfacing with metabolic maps of other organisms. We renamed the metabolites, reactions, and genes. Genes were renamed with their Entrez id.^42^ The metabolites and reactions were renamed using, if possible, the data standard from BiGG Models, a knowledgebase of genome-scale metabolic network reconstructions.^30^ Metabolites without BiGG name were renamed to their corresponding identifier in the Kyoto Encyclopedia of Genes and Genomes (KEGG) to facilitate easy lookup.^43–45^ Reactions without BiGG name were not renamed, as no standardized names exist for these reactions yet, making up 689 of not-renamed reactions.

The reactions that did not need renaming can be categorized into three groups. The first group includes transport reactions of metabolites without BiGG name. These reactions can be identified by the description of the reaction. The second group consists of reactions involved in the exchange of fatty acids between metabolites. The third group contains reactions involved in oxidation and reduction of metabolites using NADH/NAD+ or NADPH/NADP+. The second and third group kept their original annotation, linking the reaction to a KEGG entry.

## Results

In this section, we first describe the alterations in the model. These include alterations to the metabolic network, as well as the part of the model describing the relationships between genes and reactions. After that, we present the results validating our updated model. We first tested the metabolic expansion of the model by checking it for a list of metabolic functions, determining a minimal feed, and predicting mitochondrial function in respiration simulations. Next, we tested the GPRs in the model by doing knockout simulations. Finally, we apply the model to predict metabolic changes due to infection with *M. marinum*.

### Reaction network

**FIG. 2.**
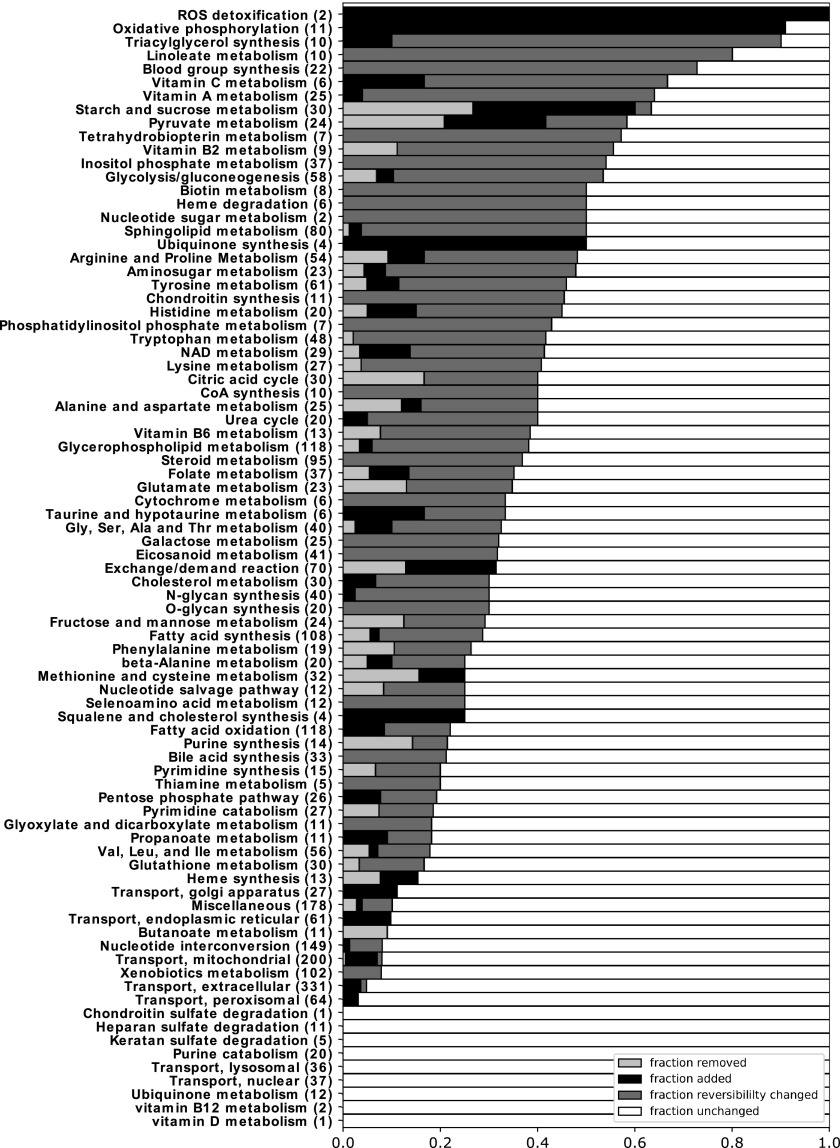
Subsystem overview of the adaptations made to ZebraGEM. For each subsystem, the total number of reactions, including the removed and added reactions, is noted in between *brackets*.

The alterations to the metabolic network encompassed the following five issues: (1) improvement of the biomass function and addition of reactions to enable synthesis of biomass precursor metabolites; (2) addition of oxidative phosphorylation; (3) correction of starch metabolism; (4) correction of the reversibility of reactions and their catalyzed or spontaneous nature; and (5) validation of the list of metabolic functions ZebraGEM was reported to be able to fulfill. Figure 2 summarizes the update in ZebraGEM, categorized into subsystems following the subsystem reaction associations from Virtual Metabolic Human (VMH), a human- and microbe-specific database on metabolism and metabolism modeling.^46,47^ The subsystems are sorted according to the number of reactions changed in each subsystem. Changes are of three types: “reaction added,” “reaction deleted,” and “reversibility changed.”

#### Biomass function and biomass precursors

FBA and related modeling approaches^7,34,48,49^ assume that an organism or cell channels the metabolic fluxes to optimize a metabolic function, called the objective function. This objective function is often a biomass function, describing the relative amounts of precursor metabolites required for biomass production. Realistic biomass functions improve the realism of model predictions.^50^ In the absence of exact data for zebrafish, we based the updated biomass function upon data from other vertebrates.

The biomass function coefficients were taken to be the average of the coefficients of biomass function of a human genome-scale reconstruction (Recon 2^18^) and a mouse genome-scale reconstruction (iMM1415^19^), so far the only other vertebrates with genome-scale reconstructions, together with Chinese hamster^20^ and rat.^23^ If a metabolite was a precursor in only one of Recon 2 and iMM1415, the coefficient was taken directly from the model in which the metabolite was present. If a metabolite was not present in both models, the coefficient was the average of a third, human three-tissue model, which had a biomass function for each tissue type.^26^

Of the biomass precursors, 14 reactants and 2 products originally had stoichiometry coefficient 0 and were put in the biomass reaction for future work. Three of the reactants were cysteine, proline, and tyrosine, and with addition of reactions to their synthesis pathways, they could be produced. Nine of the reactants were membrane lipids, like cholesterol, sphingomyelin, and phosphatidylinositol, which also could be produced after the addition of reactions involved in their synthesis. We updated their coefficients in the same way as the other metabolites taking part in the biomass function. The remaining four metabolites were NAD, NADP, NADH, and NADPH. These were omitted from the biomass function, following Recon 2, iMM1415 and the human three-tissue model. iMM1415 nor the three-tissue model contained these metabolites in their biomass function. The resulting coefficients and their origin can be found in Supplementary Table S1.

#### Oxidative phosphorylation and starch metabolism

Oxidative phosphorylation in the model is an essential pathway for respiration. The corresponding reactions and genes were added to the model, using the human metabolic model Recon 2 as a template. Along with oxidative phosphorylation, it was also necessary to update “Ubiquinone synthesis,” as well as to add the reactions CATm and SPODMm, represented in “reactive oxygen species (ROS) detoxification,” to have a functional oxidative phosphorylation pathway.

We have also revised glycogen metabolism, using Recon 2 as a template, as the stoichiometry in the original model led to mass imbalance. The original reactions were replaced with those from Recon 2, replacing the genes within the GPRs for zebrafish orthologs. Changes in glycogen metabolism are shown in Figure 2 under subsystem “Starch and sucrose metabolism” according to VHM.

#### Reaction reversibility and reaction nature

All reactions in the model were checked for reaction reversibility. This corrected two types of unrealistic behavior. First, ZebraGEM produced essential nutrients through backward reactions (Supplementary Table S2). This was solved by correcting nonbiological reversible reactions in the corresponding pathways. Second, several metabolites were tunneled over membranes, as the same reaction occurred on both sides of a membrane that involved a membrane metabolite. If at least one of these reactions was reversible, this could result in spurious transport of the nonmembrane metabolites, often NAD or NADP. By checking the reversibility of the reactions with the reaction databases BiGG, VMH, and KEGG combined, this free transport cycle could be broken. The fraction of reactions with reversibility changed per subsystem is shown in Figure 2. In total, the reversibility of 543 out of 3023 reactions was changed.

A final check was done to ensure that all reactions in the updated model do occur in zebrafish metabolism. Reactions without gene regulation were checked using the KEGG database, a database containing information on genes and reactions. Their KEGG entries were tested for two conditions: (1) whether the reaction could occur nonenzymatically, and if not, then (2) it was checked whether the reaction has an enzyme associated to vertebrates, thus excluding reactions that occur in bacteria only. If any of these two conditions was met, the reaction was kept; otherwise, we deleted the reaction. The subsystems with deleted reactions are also shown in Figure 2.

#### Metabolic functions

The original model was reported to fulfill 160 metabolic functions, ranging from amino acid metabolism to pyrimidine and purine metabolism. In our hands, using the downloadable SBML file of the original model in the supplements, only 92 of these functions were fulfilled (Supplementary Table S3). Twenty-seven of the failed functions required metabolites in compartments that were absent in those compartments in the model. The other failed functions were checked manually using From Metabolite to Metabolite (FMM^51^) and KEGG for missing reactions, or for missing transport reactions that should be present in zebrafish. The missing reactions and their corresponding genes were added to the model. An overview of the subsystems with reactions added is shown in Figure 2.

### Genes and gene-protein-reaction associations

The original model already had 2446 gene-associated reactions coded for by 4988 genes (1498 unique genes). We extended the model by putting these gene products into a GPR, and added this to the model according to the SBML guidelines. As a result, the full model can now be read and run using constraint-based modeling software, and is now suitable for gene knockout simulations and simulations with gene expression data integration.

In summary, 95 reactions were removed and 140 were added to the model, and 543 reactions had changed reaction reversibility. The updated model now contains 3023 reactions with 2810 metabolites, of which 1557 were unique, and 1636 genes. Two thousand five hundred and twenty-three reactions are gene regulated and 1678 reactions are blocked, that is, are unable to carry any flux due to dead-end metabolites. A comparison between the original ZebraGEM model and the updated model is shown in Table 1.

**Table 1.** Comparison of the Original ZebraGEM Model with the Updated Version

*No. of*	*ZebraGEM*	*ZebraGEM 2.0*
Reactions	2911	3023
Metabolites	2742	2810
Unique metabolites	1554	1557
Genes	1498	1636
Gene-regulated reactions	2446	2523
Blocked reactions	1572	1678
Successful metabolic functions	92	123
Failed metabolic functions	41	12
Metabolic functions missing metabolites	27	25

### Model validation

To check whether the changes in the model network improved the performance of the model, we tested the model predictions as follows: (1) we checked whether the model performed the metabolic functions reported in Bekaert ^22^; (2) we checked for biological validity of the minimal set of metabolites required for model growth; (3) we checked whether the model could reproduce pharmacological interference with respiration. We utilized the addition of the GPR by doing single- and double-knockout experiments, and ultimately by gene expression data integration.

#### Model metabolic functions

ZebraGEM was published with a list of 160 metabolic functions it was reported to fulfill (Supplementary table 3 of Bekaert^22^). A metabolic function on this list consists of one or multiple starting metabolites and one or more end metabolites, indicating that a metabolic route between these metabolites fulfills this function. We tested these functions by setting an import reaction for the starting metabolites and an export reaction for the end metabolites. The export reaction for the end metabolites was chosen as the objective function, and a function was deemed successful if the model imported the starting metabolites and exported the end metabolites. Some of these metabolic functions could not be tested, as the starting or end metabolite was not present in the model. Metabolic functions that did not result in a success immediately were checked by hand to see whether the model has an alternative path to fulfill the demand for the end metabolite.

Out of the 160 metabolic functions, after the corrections, ZebraGEM 2.0 was able to perform 123 functions successfully and still failed to perform 12 functions. Of the remaining 25 metabolic functions, the starting or end metabolite was absent in the model and the corresponding function could not be tested (Table 1).

#### Minimal feed composition

**FIG. 3.**
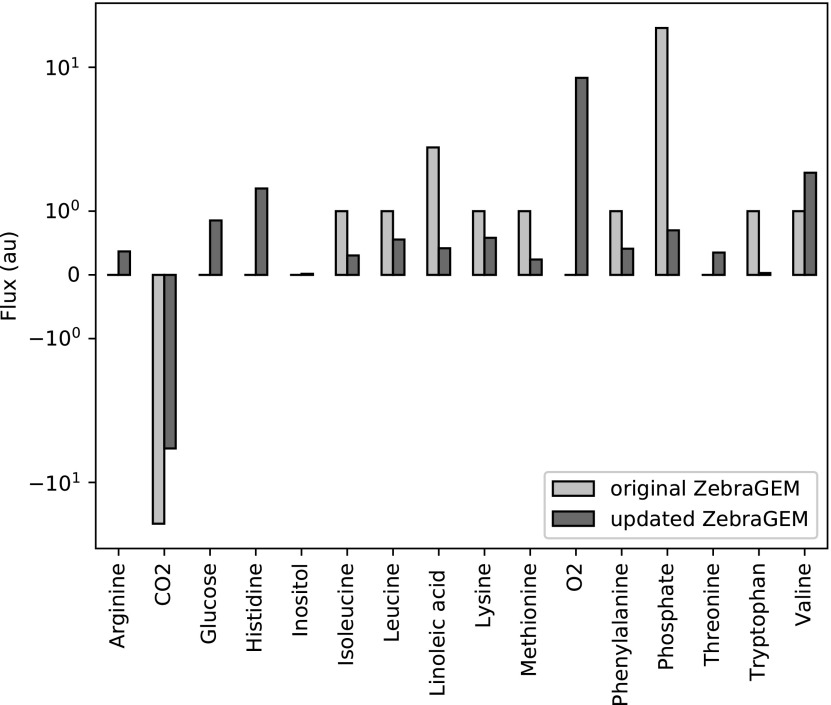
Minimal required metabolite uptake fluxes for the production of 1 arbitrary unit of biomass flux for both the original model and the updated model. Metabolite excretion fluxes are also shown, but were not constraining the minimization.

To validate the new biomass function and the changes to the reaction reversibility, which corrected spurious production of essential amino acids, we determined a minimal feed composition that would allow for growth. The model was set to produce 1 arbitrary unit of biomass flux. As the model objective, we minimized the uptake of metabolites from the environment. The source metabolites include amino acids, the fatty acids linoleic acid and linolenic acid, minerals, oxygen, and inositol (Fig. 3). We chose glucose as the sole carbohydrate source.

The updated model predicts that the amino acids arginine, histidine, and threonine are essential for biomass production, whereas they were nonessential in the original model (Fig. 3). The updated model also predicts additional uptake of glucose. In the original model, spurious glucose was produced from imbalanced glycogen reactions, leading to increased glucose uptake in the updated model. The updated model now also predicts uptake of oxygen, due to the updated model for oxidative phosphorylation (data not shown). The ratio between the metabolite species taken up from the environment has also changed in the updated model, due to the updated stoichiometry of the biomass function. This is most clearly the case for phosphate uptake (Fig. 3), which dropped from 71% of total metabolite uptake to 3%.

Thanks to the updated biomass function, inositol is now also an essential metabolite for growth in the model. Inositol is thought to be essential for zebrafish as no gene for inositol-3-phosphate synthase has been found. Inositol essentiality has been experimentally confirmed in other fish species, even in fish species with *de novo* synthesis of inositol.^52–54^ The model currently does not require the essential fatty acid linolenic acid to grow, as the lipid metabolism in the model uses a generic fatty acid and the correct conversion of linolenic acid into this generic fatty acid is not present in the model. Further improvements connecting and specifying the used fatty acid in the lipid metabolism subsystem are required; see also in the Discussion.

#### Respiration

**FIG. 4.**
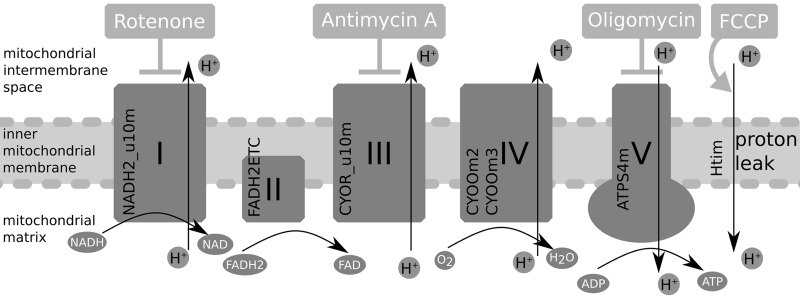
Overview of oxidative phosphorylation, with the site of action of the disrupting compounds rotenone, Antimycin A, oligomycin, and FCCP. The model reaction names are next to the corresponding enzyme, except for Htim, which represent, the proton leak and hence has no corresponding enzyme.

We next tested if ZebraGEM 2.0 correctly predicts oxidative phosphorylation. The mitochondrial oxidative function of zebrafish can be tested *in vivo* by measuring the oxygen consumption rate, which has been done in zebrafish embryos.^55^ In Gibert *et al.*,^55^ the consumption rate of oxygen has been measured under the addition of three different compounds disrupting oxidative phosphorylation. We have simulated the effects of these compounds using the updated ZebraGEM model with pFBA. The site of action of these compounds and the model reactions active in oxidative phosphorylation are shown in Figure 4.

First, the basal respiration rate is determined. In the experimental setup, this was done by measuring the oxygen consumption flux of embryos in the absence of disrupting chemicals. In our simulations, we optimize the model for biomass production with pFBA. Because the cellular environment within zebrafish is unknown, we used 1000 randomly created environments. For each of these environments, we sampled the upper bounds of metabolite uptake from selected ranges, such that the uptake was the constraining factor in biomass optimization. We used the same random environments for simulations of disruptive compounds.

**FIG. 5.**
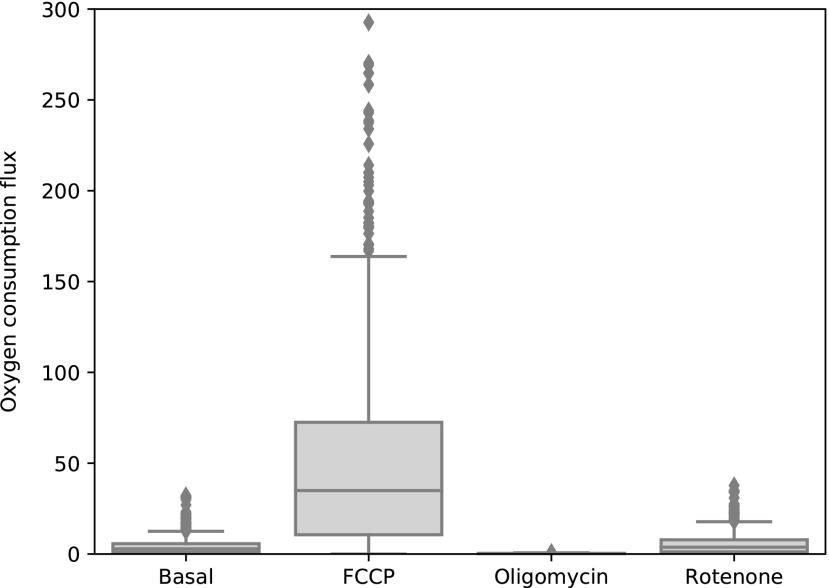
Oxygen exchange for the four modeling conditions shown in box plots.

Second, in Gibert *et al.*,^55^ the maximal respiration rate was measured after exposure to the proton uncoupler FCCP. This uncoupler allows for proton flux over the inner mitochondrial membrane, bypassing ATPase. We simulated this by blocking the model reaction ATPS4m (Fig. 4), the model equivalent of ATPase, and again optimizing for biomass production with pFBA. The experimental results show a 29% increase in respiration compared to basal respiration. Our FCCP simulations, Figure 5, second column, show a 10-fold increase in mean value compared to our basal respiration simulations mean value.

After that, a new assay was performed in Gibert *et al.*,^55^ exposing the embryos alternatively to oligomycin, an ATPase inhibitor, and rotenone, a complex I inhibitor. By comparing the respiration rate after oligomycin addition, the respiration related to ATP production can be derived. We simulated the effect of oligomycin by again blocking ATPS4m, together with limiting the flux through the uncoupling reaction that transports protons over the inner membrane (Htim, Fig. 4). The latter constraint is necessary as proton gradients cannot develop in FBA. The Htim flux upper bound was set equal to the Htim flux from the basal respiration simulations to reflect the maximal buildup of proton gradient. The experimental results show that ATP turnover-related respiration contributes about 60% to basal mitochondrial respiration; in our simulations, this would be about 90%. This is due to a side effect of blocking ATPS4m together with the limit on Htim. As the proton back flow is limited, ubiquinone cycling is also limited. Ubiquinone is required for the reaction catalyzed by dihydroorotate dehydrogenase, an essential part of pyrimidine synthesis. With limited pyrimidine synthesis, the biomass production is also limited. As the upper bound for Htim is often 0, the model does not grow at all, and hence requires no oxygen.

The final compound rotenone can be used to measure the nonmitochondrial respiration, as the electron transport chain is blocked and no oxygen is consumed by complex IV. We modeled the effect of rotenone by blocking the reaction associated to complex I: NADH2_u10m (Fig. 4). The experimental results show that nonmitochondrial respiration contributes to about 40% of basal respiration. Our simulations show a different picture, as the oxygen consumption flux is larger in the rotenone simulation than in the basal simulation. (Fig. 5, column 4). The rotenone simulation should represent respiration where the entire electron transport chain has been blocked, resulting in nonmitochondrial respiration. However, by only restricting the flux of NADH2_u10m, the electron transport chain is not entirely blocked in the model, allowing for respiration similar to the basal case. An extra compound that can be used to study nonmitochondrial respiration is Antimycin A, which inhibits complex III. Although not used in Gibert *et al.*,^55^ we tried simulating the effects by blocking the complex III corresponding reaction CYOR_u10m. However, in this case, the model fails to grow at all.

Overall, the model is able to simulate the qualitative behavior of basal, FCCP-influenced, and oligomycin-influenced respiration. It is impossible to use FBA to describe the proton gradient. Our choice to describe the proton gradient with Htim flux from the basal simulation proved too strict, and choosing a higher Htim upper bound could improve the model outcome. The rotenone/Antimycin A simulations also exposed some problems with the model that are still open, such as alternative electron transport routing and total biomass dependency on the reaction CYOR_u10m.

#### Gene-knockout simulations

Next, we validated the utility of the GPRS by performing an *in silico* screen for gene knockouts. To simulate a gene knockout, we set gene activity to “false” in each GPR that contains the gene. The other genes in the GPRs were set to “true,” and the logical expression of the GPR was evaluated. If the GPR evaluated as “false,” the flux through the associated reaction was blocked. Using FBA, we optimized biomass production in the presence of the additional constraint. The procedure was repeated for each gene. We also screened for double gene knockouts. In this case, each pair of genes in the network was set to “false” and the same procedure was applied for double knockouts. The resulting knockout biomass production rate was expressed as a fraction of the wild-type biomass production rate, that is, we divide to optimal biomass production rate in the knockout case over the optimal biomass production rate in the “wild-type” control.

Out of the 1636 genes in the model, 74 single knockouts completely blocked biomass production. For further 30 genes, single knockout reduced biomass production rates. Out of these 30 single knockouts, 13 single knockouts resulted in a biomass production rate ranging from 0.4038 to 0.8 of the optimal biomass production rate and 17 have a slightly reduced biomass production rate ranging from 0.8 to 0.95 of the optimal rate. A further 42 single knockouts resulted in a very minor reduction in biomass production, ranging from 0.95 to 0.9998 of that of the wild type. All these genes are listed in Supplementary Table S4A. The model was robust to single knockout of the 1490 other genes in the model, yielding a biomass production rate identical to that of the wild type. The genes resulting in a nonoptimal phenotype were mostly involved in oxidative phosphorylation (37 of 146), followed by cholesterol metabolism (14), nucleotide interconversion (8), and synthesis (11). We see a good correlation of the essential and partial-essential genes and the pathways for biomass precursors that we added to the biomass function as well as oxidative phosphorylation.

**FIG. 6.**
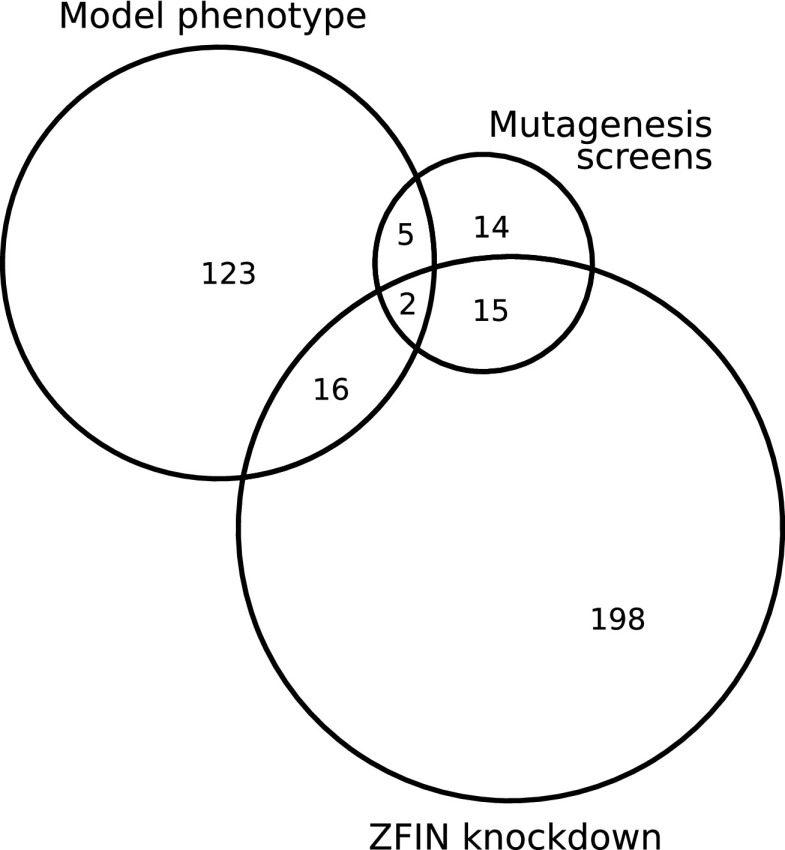
Venn diagram of genes present in the model that result in a phenotype in the single knockout simulation (model phenotype), are present in the genetic screen studies (screens),^55–62^ and have a knockdown abnormal phenotype registered in the Zebrafish Information Network (ZFIN) (knockdown).

To validate our single-gene knockout simulation results, we searched the literature for mutagenesis screens in zebrafish screening for visible defects (Fig. 6).^56–63^ Thirty-six of all our model genes had at least one record in these screens. Out of these 36 genes, 6 knockouts were among the 74 knockouts with fully blocked biomass production (*paics*, *tyms*, *cdipt*, *rrm1*, and *cad*). One knockout (*atp5po*) resulted in a reduced biomass production rate of 0.509 of the wild-type rate. For the remaining 29 knockouts from these *in vivo* screens, ZebraGEM 2.0 did not predict a reduced biomass production. These genes without model phenotype are also included in Supplementary Table S4A.

We next used ZebrafishMine to extract single-gene knockdown non-normal phenotypes from the Zebrafish Information Network (ZFIN).^64^ Around 232 genes present in ZebraGEM 2.0 had a knockdown phenotype in ZFIN. Of those 232 genes, 18 genes also had reduced biomass production in the single knockout simulations (Supplementary Table S4A and Fig. 6), 8 had no growth, 1 had rate 0.647 of wild-type rate, 5 had a rate in the range 0.8–0.95 of wild-type rate, and 4 had a rate ranging from 0.95 to 0.9998 of wild-type rate. The low number in overlap between model knockout phenotypes and *in vivo* phenotypes can be caused by open problems within the model.

On the other hand, not every gene has been extensively studied in zebrafish, which might also explain part of the model knockouts with reduced biomass production rate, but no record in the zebrafish literature. For this reason, we also used ZebrafishMine to check the remaining 123 genes that have a phenotype in the model for diseases associated with their human orthologs. Of these 123 genes, 69 have a metabolic disease associated to their human ortholog, with the exception of *sod2* and *got1* that are associated with microvascular complications of diabetes and low serum levels of aspartate aminotransferase, respectively (Supplementary Table S4A). Of the remaining 54 genes without associated disease, there is still the possibility that they point to problems in the model, or that they are associated with rare mutations that have not been studied yet. Twenty-five of these genes were related to oxidative phosphorylation, which might indicate the latter.

In total, 228 genes appeared in Refs.^55–62^ and ZFIN with a non-normal phenotype, but showed no phenotype in the single-gene knockout simulation. We categorized the effects of the knockout of these genes. One hundred and seven genes were involved in blocked reactions only, so knocking those out results in no change in the model. For 59 genes, the corresponding reactions of the genes would divert flux from the biomass production; thus, if wild-type model is optimized for biomass production, those reactions are already minimized to 0 flux. Next, there were also 42 genes that are redundant in our model: knocking those out does not block any reaction. It could be that subfunctionalization on the level of enzyme kinetics causes the *in vivo* phenotype, which cannot be represented with FBA modeling. Finally, there are 20 remaining genes that do not fit any of the three categories mentioned. Their associated reactions might be redundant within the network or do not contribute to biomass production.

For the double knockouts, we looked at two sets of genes pairs. First, we looked for pairs of genes with lower growth rates, which do not involve genes with phenotype in the single knockout simulation. The gene pairs with lowered growth rate (44 in total, 22 of which show no growth at all) are shown in Supplementary Table S4B, and are often paralogous genes. We also checked gene pairs involving at least one gene with a lowered growth rate in the single knockout experiment, which resulted in no growth, and found 36 pairs, also shown in Supplementary Table S4B. Lethal double knockouts are mainly involved in lipid metabolism, amino acid metabolism, and the citric acid cycle. In contrast to the single knockout simulation, the gene pairs that are lethal only in double knockouts do not account for much of the newly added reactions, with the exception of gene pairs involved in oxidative phosphorylation.

### Integration of expression data

Thanks to the GPRs, ZebraGEM 2.0 can predict metabolic changes driven by changes in gene expression. We demonstrate this application of ZebraGEM 2.0 with a published dataset of infection with the fish tuberculosis bacterium *M. marinum*.^65^ Briefly, zebrafish larvae were injected in the yolk with *M. marinum* at 2 h postfertilization.^65^ Gene expression in infected and control larvae was measured at 4 and 5 days postfertilization using RNA deep sequencing. This yielded a data set containing the expression of 31,388 genes.

**FIG. 7.**
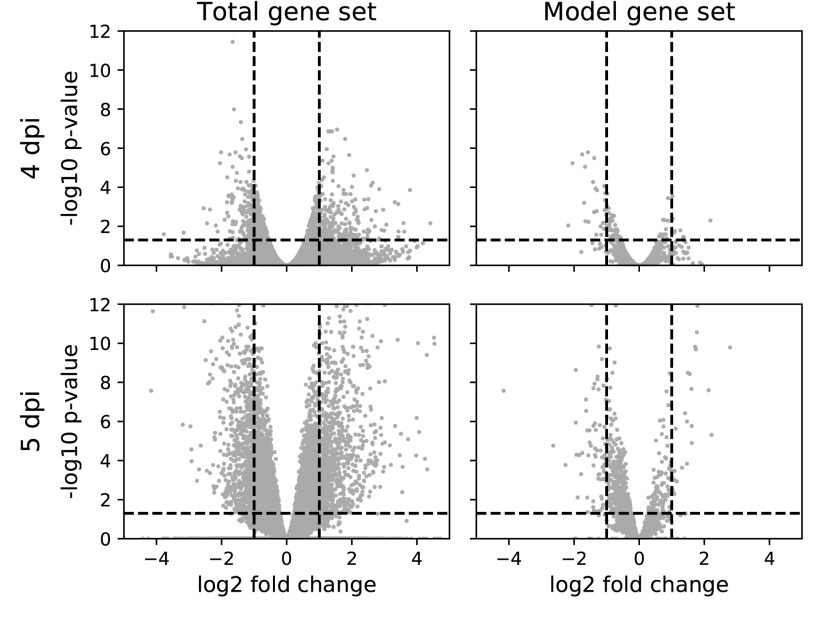
Volcano plots of the gene expression data set for both 4 and 5 dpi. Total data set on the *left*, the model subset on the *right*. *Dashed lines* indicate cutoff values: $- { \rm{lo}}{{ \rm{g}}_{10}} \left( { \rm{p}} \right)  >  1.301 , \; \left\vert {{ \rm{lo}}{{ \rm{g}}_2} \left( {{ \rm{fold \;change}}} \right) } \right\vert  >  1$. dpi, days postinfection.

Of these 31,388 genes, 1608 genes are present in ZebraGEM 2.0. Although this is a small fraction of the total gene set, it covers 98% of the model genes. From these 1608 genes present in ZebraGEM 2.0, we selected genes with differential expression in the infected and control groups at 4 and 5 days postinfection (dpi). Genes were considered “differentially expressed” if they had a fold change $> 2$ or a fold change $< - 2$, together with an adjusted *p*-value threshold $< 0.05$ (Fig. 7). We thus identified 24 metabolic genes in ZebraGEM 2.0 that were differentially expressed both at 4 dpi and 5 dpi (Tables 2, and 3).

**Table 2.** Number of Differentially Expressed Genes in the Total Gene Expression Dataset and the Subset of Genes Present in the Model

	*Total gene set*	*Model gene set*
4 dpi	408	35
5 dpi	1714	106
Both dpi	226	24

dpi, days postinfection.

**Table 3.** List of Genes Differentially Expressed at Both 4 and 5 dpi That Are Present in the Model

*Gene symbol*	*Gene name*
*acsl5*	Acyl-CoA synthetase long-chain family member 5
*ampd3b*	Adenosine monophosphate deaminase 3b
*anpepb*	Alanyl (membrane) aminopeptidase b
*asah2*	N-acylsphingosine amidohydrolase 2
*dpys*	Dihydropyrimidinase
*elovl8b*	ELOVL fatty acid elongase 8b
*enpp7.1*	Ectonucleotide pyrophosphatase/phosphodiesterase 7, tandem duplicate 1
*ftcd*	Formimidoyltransferase cyclodeaminase
*gch2*	GTP cyclohydrolase 2
*ggt1b*	Gamma-glutamyltransferase 1b
*mboat2a*	Membrane bound O-acyltransferase domain containing 2a
*neu3.3*	Sialidase 3 (membrane sialidase), tandem duplicate 3
*neu3.4*	Sialidase 3 (membrane sialidase), tandem duplicate 4
*pfkfb3*	6-Phosphofructo-2-kinase/fructose-2,6-biphosphatase 3
*ptgs2a*	Prostaglandin-endoperoxide synthase 2a
*sat1a.2*	Spermidine/spermine N1-acetyltransferase 1a, duplicate 2
*slc13a3*	Solute carrier family 13 (sodium-dependent dicarboxylate transporter), member 3
*slc26a3.2*	Solute carrier family 26 (anion exchanger), member 3, tandem duplicate 2
*slc7a7*	Solute carrier family 7 (amino acid transporter light chain, y+L system), member 7
*tdo2a*	Tryptophan 2,3-dioxygenase a
*tyms*	Thymidylate synthetase
*ugt1ab*	UDP glucuronosyltransferase 1 family a, b
*uroc1*	Urocanate hydratase 1
*zgc:92040*	zgc:92040

We next predicted the metabolic changes caused by differential expression of these 24 expressed genes. We made use of GC-flux.^37^ The GC-flux algorithm constrains the rate of the metabolic reaction in the model based on the expression levels of the genes coding for the corresponding enzymes. GC flux distributes the gene expression of a single gene over all reactions associated with that gene, such that the total sum of those reaction fluxes cannot exceed maximum flux associated with the gene expression value. We performed this analysis for control and infected larvae at 4 and 5 days dpi.

After the model was constrained with the gene expression data, a method called FVA was applied.^8^ FVA predicts the minimum and maximum possible flux ranges for each reaction, given an objective function; in this study, we used biomass production rate. To compare the flux ranges between control infected at 4 and 5 dpi, we used the RFRC.^39^ The RFRC is a measure that indicates how much the flux ranges differ between the control and infected simulations. When the RFRC is greater than 1 or smaller than −1, the centers of the compared flux ranges are separated by more than the averaged width of those flux ranges, with negative values indicating that the infected case has a range lower than the control case.

An important reaction with an absolute RFRC greater than 1 is the biomass function BIO_L_2 and it appears in the list for both 4 and 5 dpi. The RFRC of BIO_L_2 is negative in both cases, −18.371 for 4 dpi and −17.421 for 5 dpi, suggesting that infection reduces biomass production rate. When comparing the maximal growth rates, the growth rate of the infected simulation was 83% of the control growth rate at 4 dpi, and at 5 dpi, the infected group reached 84% of the growth rate of the control. Further examination of the list with reactions with absolute RFRC greater than one (Supplementary Table S5) shows that affected reactions (with $\left\vert {RFRC} \right\vert > 1$) at 5 dpi (46 reactions in total) are also affected at 4 dpi (56 reactions in total). Most of these 46 reactions were essential reactions involved in biomass precursor production and their knockouts are lethal (Supplementary Table S4A). The fluxes of the biomass precursor reactions co-vary, because they contribute, often in parallel, to the biomass reaction. If one of the fluxes is reduced, biomass production rate is also reduced. Due to flux balance, all the other biomass precursor fluxes must be reduced as well.

To gain insight in which genes give rise to such restricting reactions, and hence are limiting growth in our simulations, we identified the genes that restricted biomass production by comparing the flux corresponding to each gene with the expression level of each gene (Table 4). In total, 17 genes restricted biomass production in at least one of the four cases (condition x dpi). Aside from essential biomass precursor reaction-associated genes (essential genes for the model), 9 genes out of 17 are not essential to the model. Among these are *si*:*ch1073*-*100f3*.2, *slc5a9,* and *tha1*, all associated to monosaccharide transporters. The differential expression of *slc2a11a,* also associated to a monosaccharide transporter, together with limited availability of flux for the other monosaccharide transporters, puts a large restriction on the model. The low number of only four genes with differential expression (namely *ftcd* at both 4 and 5 dpi, and *gck*, *nme4*, and *slc2a11a* at 5 dpi only) points toward a drawback of this data integration method: it only looks at the mean values of each case, but ignores whether these means are significantly different.

**Table 4.** Genes with Gene Expression Restricting Biomass Production in the Model with Their Fold Change and Their Essentiality Within the Model, According to Lethal Phenotypes (Essential) and Reduced Growth Phenotypes (Semiessential) in Supplementary Table S4A

*Gene*	*FC 4 dpi*	*FC 5 dpi*	*Essentiality*
acacb	0.522	0.036	Essential
arg1	−0.402	−0.837	Semiessential
atp5s	0.358	0.088	Semiessential
bdh2^*^	−0.403	−0.810	Semiessential
cox6a2	−0.437	−0.633	Essential
*ftcd*	***−1.061***	***−1.353***	***Semiessential***
galk1	−0.173	−0.669	—
galk2	−0.314	−0.315	—
gart	−0.262	−0.016	Essential
**gck**	**1.871**	**−4.162**	—
hkdc1	−0.529	−1.469	—
**nme4**	**−0.548**	**−1.147**	—
nme6	−0.548	−0.267	—
si:ch1073-100f3.2^*^	−0.492	−0.277	Semiessential
**slc2a11a**	**0.068**	**−1.014**	—
slc5a9	−0.788	−0.791	—
tha1^*^	0.489	0.686	—

Genes marked with an *asterisk* are not restrictive for 5 dpi. *Bold face* genes have differential expression for 5 dpi, *bold and italic* font both 4 and 5 dpi.

FC, fold change.

We observed that there was a reduction in growth rate in the infected case, and could ascribe this to a number of restricting genes. However, growth reduction might not be the only difference in metabolic activity; which metabolic pathways are contributing to biomass production can also differ between control and infected. To see if there was also a shift in which metabolic pathways contribute to biomass production, the flux ranges were normalized with the biomass flux. The RFRC was then again computed with the normalized ranges, and only for 4 dpi were there reactions with |RFRC| *>* 1. These reactions are HISD, IZPN, URCN, and EX_his__L_e, and are involved in the pathway converting histidine into glutamate. The high |RFRC| of these reactions can be directly linked to the differential expression of *uroc1*.

Overall, the addition of GPRs to ZebraGEM 2.0 together with GC-flux allowed us to integrate gene expression data into ZebraGEM 2.0, providing us with novel insights into potential metabolic changes due to *M. marinum* infection. First of all, there is a reduction in growth in the infected cases. This can be attributed to differences in the expression of some essential genes as well as monosaccharide transporter genes. When looking at qualitative changes in metabolism, histidine metabolism is reduced at 4 dpi, due to reduced expression of *uroc1.* Together with the restrictive gene *ftcd* (Table 4), which is also involved in the histidine pathway, this could make the histidine pathway an interesting starting point for more research on changes in metabolism upon *M. marinum* infection.

## Discussion

In this work, we have presented ZebraGEM 2.0, an improved version of the genome-scale metabolic reconstruction ZebraGEM.^22^ We have made the model available through an xml-file, see Supplementary Data. The improvements were the addition of GPRs, significant changes to the stoichiometry by the addition of oxidative phosphorylation and checking the reversibility of reaction, and adhering to the existing standards of genome-scale metabolic reconstructions. To validate the new model, we have shown that it performs better than the previous version on a predetermined list of 160 metabolic tasks. We also determined a minimal feed. ZebraGEM assigns more nutrients to be essential, which is in agreement with what is known about zebrafish nutrition. To test the added GPRs, we did an *in silico* knockout screening, and found a large agreement between genes causing a phenotype in the model and genes that are known to have a phenotype *in vivo* in zebrafish or in human.

Altogether, ZebraGEM 2.0 is now suitable to be used with gene expression, which we demonstrated by integrating a gene expression data set of *M. marinum-*infected and noninfected embryos. In this study, our simulations predicted a lowered growth rate for the infected embryos due to changes in essential gene expression as well as monosaccharide transporter gene expression, and a change in histidine metabolism.

Here, we will discuss further improvements and limitations of ZebraGEM 2.0, and briefly discuss the future work.

### Blocked reactions

Blocked reactions are reactions that cannot carry any flux due to absence of some or all pathways carrying metabolites toward or away from the reactions. Currently, 1675 out of 3018 (55.5%) of the reactions remain inactive in ZebraGEM 2.0. This number is high in comparison with similar metabolic reconstructions: in Recon 2, 2123 out of 7440 (28.5%) reactions are blocked,^18^ and in iMM1415, 1294 out of 3726 (34.7%) reactions are blocked.^19^ Even if the blocked reactions are currently nonfunctional, we have decided to leave them in ZebraGEM 2.0. This prepares the model for future improvements that can unblock these reactions.

To unblock these reactions, we will need to add a number of missing exchange reactions. These allow the model to import metabolites and excrete waste metabolites. Due to flux balance, the whole metabolic pathway is blocked if excretion or further processing of a metabolite is impossible. One example of such a missing exchange reaction is the exchange reaction for urea; after we added it to the model, it allowed for the production and incorporation into biomass of arginine. For our current needs, further addition of exchange reactions was not needed. Besides that, improvements in the import and export reactions are complicated by three facts. First, there is the food composition, which is not predetermined for free-feeding larvae and adult fish; a solution here would be to add all possible exchange reactions and open or close them depending on fodder composition. Second, there is the unknown factor of exchange with the environment by other means than diet, such as excretion and uptake of metabolites through the skin. Third, there is exchange among cells and tissues of metabolites, such as the uptake of nutrient from the yolk in developing embryos.

Further unblocking of reactions will be achieved by identifying unconnected parts of the network and add the missing metabolic pathways. Such gap-filling can, in part, be automated by finding the minimal set of addition to the network,^66–68^ or using novel topology-based methods that can pinpoint missing essential reactions.^69^ Such automized gap-filling should be done with care, because the gaps often require reactions that have no or little literature that clearly supports those reactions.

### Lipid metabolism

ZebraGEM 2.0 and its predecessor have applied a number of simplifications in the description of lipid metabolism. First, a generic fatty acid is used in most lipid metabolism reactions. Also, the essential lipid linolenic acid has no reaction in the model converting it into this generic fatty acid and hence is not processed further by the model. To further improve the description of lipid metabolism in ZebraGEM 2.0, future description of lipid metabolism should include specific reactions for each type of fatty acid. This improvement would make linolenic acid essential, but because a single reaction would be part of the metabolism of a range of fatty acids, it comes at the cost of increased model size. Most likely, this will double the number of reactions, as the ∼600 reactions involved in lipid metabolism will be multiplied by the number of specified fatty acids. This will increase simulation time significantly for some of the modeling techniques, like FVA. The Chinese hamster model iCHOv1,^20^ a human platelet model,^70^ and a human erythrocyte model^24^ have parts of lipid metabolism with specified fatty acids and can serve as examples.

An additional factor in lipid metabolism is that many of the associated metabolites are located in the compartment “membrane.” This compartment accounts for the plasma membrane, Golgi membrane, endoplasmic reticulum membrane, lysosome membrane, nuclear membrane, and the outer mitochondrial membrane all at once. This compartmentalization into a single compartment does not take into account the required transport processes and associated metabolic processes for such metabolites that take place within the cell. Another effect of this membrane compartment is the tunneling of NADH and NADPH over the membrane due to imbalanced reaction reversibility, as discussed in Reaction Reversibility and Reaction Nature section. We have currently solved this issue by checking reaction reversibility, but a future improvement of the compartmentalization of membrane metabolites into specific membrane parts would solve these problems more accurately.

Improving lipid metabolism is also of interest when looking at the growth conditions of zebrafish. Embryos rely on the abundance of lipids present in the yolk as their source of energy, and as zebrafish are often used for experiments in their embryonal stages, insight into lipid metabolism is relevant. Fraher *et al.* determined changes in lipid composition of both the yolk and the developing embryo.^71^ This study provides interesting information upon which estimates for lipid exchange between embryo and yolk can be made, which can further improve metabolic modeling studies of embryonic stages.

### Biomass function and quantitative simulations

The current biomass function is not based upon any data on zebrafish cell composition, but on human and mouse models. Although the metabolites of which a cell consists vary little between animals, as all cells are built from amino acids, nucleic acids, and fatty acids,^50^ the ratios between the required metabolites can vary as much as 30 million fold.^26^ The ratios of biomass precursor metabolites can have a large impact on the model predictions. Therefore, data of zebrafish cell composition, possibly for different cell types, will be of high value for increasing model prediction accuracy. So far, there has been detailed study of lipid composition only.^72^

Genome-scale metabolic modeling focuses only on metabolism and hence has a limited scope. For example, 20 genes with a non-normal phenotype in Refs.^55–62^ or ZFIN had no phenotype in ZebraGEM 2.0. They could not be ascribed to blocked reactions, no knockout effect due to the gene being redundant in the model, or the associated reaction diverting flux from the biomass optimization. The optimization for biomass production rate does likely not reflect all the required metabolic outputs of a cell. Alternative objective functions would include specific protein synthesis for antibody producing B-lymphocytes, ATP synthesis for muscle cells, or ROS production upon infection. In addition, bacterial metabolism also plays a role during infection. Therefore, results of *in silico* knockout experiments will deviate from the results of *in vivo* experiments.

A generic problem of flux balance analysis is that it does not consider kinetics and thermodynamics. Gene mutations or knockouts can change the kinetics of metabolic reactions, causing for instance accumulation of toxic compounds. Thermodynamics can also affect the rate of reactions and has been combined with constraint-based methods before.^73^ Finally, these genes can cause a phenotype *in vivo* by other means than metabolism, that is, they could be involved in signaling and genetic regulating processes as well, and those aspects are not part of this model.

Last but not least, when using data integration methods, one has to be careful with the distribution of experimental values. As we saw now with our data-integrated simulations, most of the restricting genes were not significantly differentially expressed, which could lead to pinpointing incorrect causes of altered metabolism. The algorithm we used, as well as many others take only a single value for the expression of genes, often just the average; the original distribution underlying that average has to be considered, especially when comparing different situations. Extending data integration methods for constraint-based metabolic modeling with methods from robust optimization can offer a framework in which such distributions can be taken into account.

Despite these limitations, the improved model combined with the zebrafish embryo data results in the prediction of lowered growth in the case of *Mycobacterium* infection. Furthermore, we showed that metabolism of histidine synthesis was decreased in infected zebrafish embryos. Further improvements on the model as well as the data integration methods and analysis can lead to new applications of ZebraGEM 2.0, such as elucidating yolk and embryo metabolism or exploring the causes of metabolic diseases.

## Supplementary Material

Supplemental data

Supplemental data

Supplemental data

Supplemental data

Supplemental data

Supplemental data
